# Kernig's Sign

**Published:** 1904-10-08

**Authors:** 


### KERNIG'S SIGN.

Though it is now many years since this sign was first described, its value even yet does not seem to have received general recognition. To test for it the patient is made to sit up, and an attempt is then made to extend the leg on the thigh. If the sign is present, this extension will be found difficult or impossible in consequence of spasm of the hamstring muscles. The test can also be applied with the patient recumbent by flexing the thigh to a right angle with the abdomen and then endeavouring to extend the leg. Numerous observers have reported the presence of Kernig's sign in the great majority of cases of meningitis in which the spinal meninges were affected. Thus it has been noted in 53 out of 60 instances of cerebro-spinal fever. It is also often present, but by no means invariably, in tuberculous meningitis, in which disease, though the cerebral symptoms mainly occupy attention, the pathological process very frequently extends to the coverings of the spinal cord. In a series of cases of posterior basic meningitis in children published by Dr. Hugh Thursfield,[1] Kernig's sign was present in every instance. There have, however, been a number of observations tending to show that a spinal meningitis, or, for the matter of that, even a cerebral meningitis, is not essential to the presence of Kernig's sign. The truth of the former of these propositions is afforded by the fact that it has been obtained in instances of pneumococcic and streptococcic meningitis, where the inflammatory signs were restricted entirely to the cranial cavity. Kernig's sign, apart from meningitis, has been noted by several observers. Thus Dr. W. Thyne,[2] described it in a case of cerebellar hæmorrhage, the patient being a young woman, and the symptoms also including opisthotonos and exaggeration of the tendon jerks. Dr. Thursfield, in the article already quoted, noted the presence of Kernig's sign in two instances of lateral sinus thrombosis, the absence of meningitis being guaranteed by post-mortem examination. In a case reported by Sailer,[3] Kernig's sign was present in the left lower limb only. The patient was a man of 20 years, who exhibited general signs of septic poisoning, including ulcerative endocarditis, and with these some evidences of spasm in the limb of the left side, and on this side also, both Kernig's and Babinski's sign. The autopsy revealed an old osteomyelitis of the tibia, which was the probable source of the septicæmia, and also congestion of the cerebral meninges, and a softened area in the right motor convolutions. The same author[4] reports another instance in which Kernig's sign was present only on one side, and here also muscular spasm and Babinski's sign were observed on the same side. Hence it seems probable that this sign may indicate, among other things, a partial interruption of the motor pathway on the side of the brain opposite to the limb in which it is manifested. In reference to the relation of Kernig's sign to meningitis, its discovery has sometimes led to the recognition of that disease when previously there had been no suspicion of its presence. Thus, as described by Netler,[5] a typical case of typhoid fever showed Kernig's sign, though there were no other symptoms of that condition, and the existence of meningitis was discovered at the autopsy. MM. Widal and Merklen[6] report a case of meningeal hæmorrhage with Kernig's sign. In this instance there was observed what may be called the extreme type of the sign—that is, that so long as the patient was lying down the legs were extended on the thighs, but so soon as he sat up the knees became flexed and the legs could not be brought into a straight line with the thighs. Lastly may be noted an observation made by Dr. W. J. Buchanan[7] that a phenomenon corresponding to Kernig's sign may in some instances be observed in the upper limbs—that is, spasm of the flexors, rendering it impossible to extend the forearm on the upper arm.

[1] Lancet, Feb. 16, 1901. [2] Ibid, Feb. 9, 1901. [3] Ibid, June 28, 1902. [4] Brit. Med. Jour., Sept. 20, 1902. [5] La Semaine Médicale, 1898. [6] Med. Rev., Vol. III., p. 98. [7] Ibid, p. 314.

